# The gut microbiota and aging: interactions, implications, and interventions

**DOI:** 10.3389/fragi.2025.1452917

**Published:** 2025-05-14

**Authors:** Despoina Gyriki, Christos G. Nikolaidis, Eugenia Bezirtzoglou, Chrysa Voidarou, Elisavet Stavropoulou, Christina Tsigalou

**Affiliations:** ^1^ Master Program in “Food, Nutrition and Microbiome”, Department of Medicine, Democritus University of Thrace, Alexandroupolis, Greece; ^2^ Internal Medicine Department, Vostaneio-General Hospital of Mytilene, Mytilene, Greece; ^3^ Department of Agriculture, University of Ioannina, Arta, Greece

**Keywords:** gut microbiota, gut microbiome, dysbiosis, aging, age-related diseases, longevity, probiotics, diet

## Abstract

The human microbiota, a complex ecosystem of microorganisms inhabiting various body sites, particularly the gut, plays a crucial role in maintaining health and influencing disease susceptibility. Dysbiosis, characterized by alterations in microbial composition and diversity, has been implicated in numerous diseases, including those associated with aging. This review examines the complex relationship between gut microbiota and aging, highlighting the age-associated gut microbiota alterations, the factors contributing to these changes, the links between microbiota and age-related diseases, and the potential of interventions targeting the microbiome to extend lifespan and improve health outcomes in the elderly. Further research is needed to unravel the intricate mechanisms underlying the interplay between the microbiome and aging, paving the way for innovative strategies to promote healthy aging.

## 1 Introduction

Since the early 1900s, when researchers discovered numerous microorganisms—including bacteria, yeasts, and viruses—in various parts of the human body, the human microbiota has intrigued scientists (Quigley, 2011). The term “microbiota” refers to the community of living microorganisms that inhabit a particular environment (Hou et al., 2022). The gut microbiota is the most extensive component, comprising more than 1,000 species of microorganisms. The highest density is found in the colon, which, according to recent estimates, hosts more than 3.9 × 10^13^ microbial cells (Sender et al., 2016). To a lesser extent, the human microbiota is also present in other areas, including the oral cavity, lungs, vagina, and skin.

Over the past decades, the field of human microbiota has attracted great scientific interest because of its critical influence on health and disease. Often referred to as “the hidden organ,” the gut microbiota forms a symbiotic relationship with the intestinal epithelium in healthy individuals, showcasing vital metabolic, immunological, and protective functions (Gyriki et al., 2024). For example, these microorganisms metabolize dietary components, xenobiotics, and drugs, while producing short-chain fatty acids (SCFAs), vitamins (such as K, B12, biotin, folic acid, and thiamine), secondary bile acids, and antimicrobial peptides (including defensins, cathelicidins, and lectins). They also provide antimicrobial protection by producing lactic acid and mucin and by stimulating the innate immune system and immunoglobulin A secretion (Jandhyala et al., 2015).

Αlterations in the composition and diversity of the gut microbiota, known as dysbiosis, are implicated in numerous diseases, including irritable bowel syndrome, inflammatory bowel disease, allergies, diabetes, and obesity (Brüssow, 2020; Dogra et al., 2020). Dysbiosis has also been related to aging, an unavoidable, irreversible process characterized by noticeable changes in an organism’s physical appearance and function (Hou et al., 2019). The proportion of the European population aged 65 and over increased from 14.9% in 1996 to 19.2% in 2016, with forecasts indicating a rise to 29% by 2070 (Cristea et al., 2020). As life expectancy rises, aging populations face declines in both physical and cognitive abilities, often experiencing multiple health conditions. This demographic shift places significant strain on global healthcare systems (Chen et al., 2023).

López-Otín et al. identified a set of biological processes contributing to the aging of cells, tissues, and the entire organism (López-Otín et al., 2013). A decade later, these were expanded to include twelve key hallmarks of aging (López-Otín et al., 2023). They include genomic instability, telomere shortening, epigenetic changes, loss of proteostasis, disrupted nutrient sensing, mitochondrial dysfunction, cellular senescence, stem cell depletion, impaired intercellular communication, macroautophagy, chronic inflammation, and dysbiosis. These hallmarks provide a foundational framework for understanding the aging process and are deeply interconnected. For instance, dysbiosis and chronic inflammation are closely linked, as the altered gut microbiota in elderly individuals is associated with increased intestinal permeability and immune system activation, resulting in chronic inflammation.

Given the gut microbiota’s significant impact on health and its contribution to aging, understanding its alterations in elderly individuals and its complex association with age-related diseases could provide valuable insights. Understanding these complex interactions may reveal strategies for modulating and potentially slowing down aging through gut microbiota interventions. The current review explores the existing literature on the relationship between gut microbiota and aging, examines age-associated conditions, and proposes the potential of novel microbiota-targeting interventions for extending lifespan. However, further research is needed to illuminate the intricate interplay between the microbiome and aging.

## 2 Shaping of the gut microbiota across age

**FIGURE 1:**
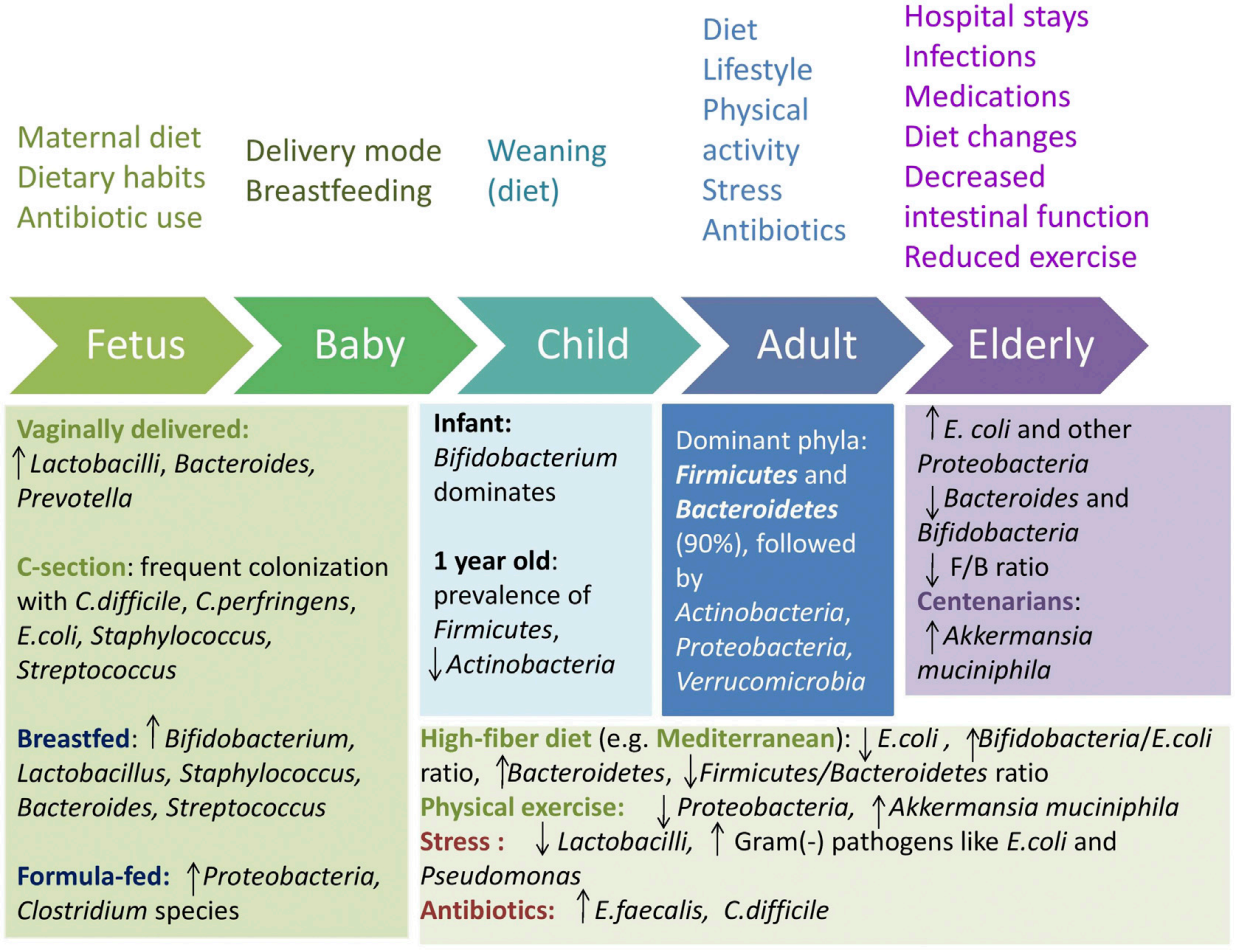
Gut microbiota shaping across age and influencing factors. F/B = Firmicutes/Bacteroidetes. Upward arrows indicate an increase and downward arrows indicate a decrease.

The gradual colonization of the gut by microbes may begin during the prenatal period, through a unique microbiota present in the placenta and amniotic fluid (Collado et al., 2016). Factors pertaining to the mother, such as health, dietary habits, and exposure to antibiotics, can influence the fetal microbiome during pregnancy (Kumbhare et al., 2019). Postnatally, factors like delivery mode, breastfeeding, and diet further shape the microbiota, leading to a relatively stable composition by the age of 3–4 years (Nagpal et al., 2017; Kumbhare et al., 2019), though some suggest that it is not fully established until the age of 5 (Cheng et al., 2016; Figure 1).

Vaginally born infants possess a distinct microbiota, with early and enhanced colonization by Lactobacilli, *Bacteroides,* and *Prevotella*, similar to the maternal vagina, whereas infants delivered via caesarean section often exhibit delayed or reduced carriage of *Bacteroides*, Bifidobacteria, and Lactobacilli and are more frequently colonized with *Clostridium difficile*, *Clostridium perfringens*, *Escherichia coli*, *Staphylococcus*, and *Streptococcus*, similar to the maternal skin and the hospital environment (Nagpal et al., 2016; Moore and Townsend, 2019). Breastfeeding promotes high levels of *Bifidobacterium*, aiding oligosaccharide metabolism found in breast milk, *Staphylococcus, Bacteroides, Streptococcus,* and *Lactobacillus*. In contrast, infants fed with a formula show a higher prevalence of Proteobacteria and *Clostridium* species (Stewart et al., 2018; Ma et al., 2020).

A major change in microbiota diversity occurs between 9 and 18 months, including an increase in Bacteroidetes, and a decrease in *Lactobacillus* spp. and Enterobacteriaceae (Bergström et al., 2014). At the age of 9–36 months, an increase in butyrate-producing *Clostridium leptum, Eubacterium hallii,* and *Roseburia* has been observed (Bergström et al., 2014). By age 1, *Akkermansia muciniphila*, *Bacteroides, Veillonella, Clostridium coccoides* spp., and *Clostridium botulinum* spp. are prevalent (Tidjani Alou et al., 2016). Actinobacteria abundance significantly declines after weaning and keeps decreasing with age (Odamaki et al., 2016), with Firmicutes becoming the most dominant phylum.

The onset of weaning signals a gradual transition of the infant gut microbiota toward that of adults, which highlights the determining role of diet on microbiota composition (Claesson et al., 2012). Comparative studies reveal that dietary habits profoundly shape the gut microbiota. African children on a high-fiber diet have increased Bacteroidetes and decreased Firmicutes, with higher levels of short-chain fatty acids and unique bacteria like *Prevotella* and *Xylanibacter*, unlike European children on a western diet (De Filippo et al., 2010). Similarly, Bangladeshi children, whose diet is rich in carbohydrates and rice, exhibit a prevalence of *Prevotella*, while US children consuming more meat and protein show a prevalence of *Bacteroides* (Lin et al., 2013).

In healthy adults, the dominant phyla are Firmicutes and Bacteroidetes, which constitute over 90% of the intestinal flora, followed by Actinobacteria, Proteobacteria, and Verrucomicrobia (Claesson et al., 2011; Cheng et al., 2016; Rinninella et al., 2019). Aging further alters the gut microbiota, with the elderly showing increased *E. coli* and other Proteobacteria and decreased beneficial anaerobes like *Bacteroides* and Bifidobacteria (Mariat et al., 2009; Biagi et al., 2010). The oldest-old typically have lower Firmicutes levels and higher Bacteroidetes levels, with the Firmicutes/Bacteroidetes ratio rising during adulthood and decreasing in older age (Mariat et al., 2009; Odamaki et al., 2016; Vaiserman et al., 2020). An increase in genera like *Akkermansia* has also been observed in centenarians, potentially linked to longevity (López-Otín et al., 2023).

Extended adherence to plant-based diets is linked to a more diverse and abundant phylogenetic composition of fecal microbiota compared to Western diets (Tsigalou et al., 2021). The Mediterranean diet, characterized by high consumption of fruits, vegetables, and legumes, has been demonstrated to be a healthy diet pattern that is beneficial for the gut microbiota. A study found that *Lachnospira* and *Prevotella* were higher in individuals on plant-based diets, whereas *L-Ruminococcus* was linked to omnivorous diets (Filippis et al., 2016). The same study showed that the Mediterranean diet had a positive impact on fecal SCFA levels, likely reflecting the presence of Firmicutes and Bacteroidetes that degrade indigestible carbohydrates. Strong adherence to this diet has also been associated with lower *E. coli* levels, increased Bifidobacteria/*E. coli* ratio, which is regarded as a crucial marker of gut microbiota balance and overall health (Mitsou et al., 2017), and higher levels of Bacteroidetes, with a lower Firmicutes/Bacteroidetes ratio (Garcia-Mantrana et al., 2018).

Lifestyle and environmental factors, such as physical activity, stress, smoking, and traveling, also influence the gut microbiota (Conlon and Bird, 2014). Athletes and individuals with a low body mass index seem to have higher levels of *A. muciniphila* compared to those with a high body mass index, which has a positive effect on metabolic health (Clarke et al., 2014). Six weeks of guided endurance exercise training has been shown to positively affect the gut microbiota by increasing the abundance of *A. muciniphila* and decreasing the abundance of Proteobacteria (Munukka et al., 2018). Daily exercise in the elderly can alleviate age-related gut microbiota differences by increasing Actinobacteria and decreasing Cyanobacteria levels (Zhu et al., 2020). Stress reduces the number of Lactobacilli, while it promotes the growth of gram-negative pathogens such as *E. coli* and *Pseudomonas* (Lutgendorff et al., 2008). Smoking, another lifestyle factor, impacts the gut microbiota by raising *Bacteroides-Prevotella*. Other environmental factors, like exposure to infectious gastrointestinal diseases while traveling, may have long-term effects on gut microbiota and health. Additionally, traveling and shift work can disrupt the circadian rhythm, further influencing the gut microbiome (Conlon and Bird, 2014).

Antibiotics significantly disrupt the gut microbiota by reducing the overall diversity, enhancing the growth of opportunistic organisms like *Enterococcus faecalis,* and elevating the risk of *C. difficile* infection (Coman and Vodnar, 2020; Ramirez et al., 2020). The impact varies with antibiotic type, dosage, and duration. Broad-spectrum antibiotics can disrupt the balance between Firmicutes and Bacteroidetes (Rinninella et al., 2019). For instance, a 7-day treatment with broad-spectrum β-lactam antibiotics doubled the microbial load in patients’ fecal samples and increased the ratio of Bacteroidetes/Firmicutes (Panda et al., 2014), while macrolides decreased Actinobacteria and increased *Bacteroides* and Proteobacteria levels (Iizumi et al., 2017).

Other medications, including osmotic laxatives, hormones, benzodiazepines, antidepressants, antihistamines, inflammatory bowel disease medications, proton pump inhibitors (PPIs), metformin, and statins, also significantly influence gut microbiota composition (Falony et al., 2016; Hou et al., 2022). Research indicates that PPIs, a commonly used medication, are associated with notable microbiota changes. For example, 20% of bacterial taxa are substantially altered in PPI users compared to non-users. Families like Bifidobacteriaceae, Ruminococcaceae, and Lachnospiraceae are often reduced, while others like Actinomycetaceae and *Staphylococcus* spp. may increase (Imhann et al., 2017; Zhang et al., 2023). Also, PPIs impose a great risk of susceptibility to infections such as *C. difficile*, *Salmonella*, and *Campylobacter*, by reducing gastric acidity (Tian et al., 2023). The stomach’s acidic environment—primarily maintained by hydrochloric acid—creates a low pH that effectively kills many pathogens that might otherwise survive and increase the risk of infections.

## 3 Age-related alterations in the composition and function of the gut microbiota

Age-related alterations in the composition and function of the gut microbiota have been extensively studied, although results vary due to differences in study populations, sample sizes, methodologies, and designs. Next, we will mention several studies from the existing literature on intestinal microbiota alterations with increasing age (Table 1).

**TABLE 1:** Summary of studies on gut microbiota alterations across age.

Study	N	Participants	Main Findings
Woodmansey et al. (2004)	28	12 adults (19-35 yo) 6 healthy elderly (67-75 yo) 10 elderly hospitalized patients receiving antibiotics (73-101 yo)	A reduction in *Bacteroides* levels and species diversity was observed in elderly groups. A similar reduction and shift were seen with *Prevotella* species. Bifidobacteria species and diversity decreased in elderly, especially in hospitalized patients. Fusobacteria, Propionibacteria, and Clostridia increased in antibiotic-treated elderly. Short-chain fatty acids decreased with increased age.
Mariat et al. (2009)	62	21 infants (3 weeks-10 months old) 21 adults (25-45 yo) 20 elderly (70-90 yo)	Infants: Bifidobacteria was the most abundant group. Four dominant bacterial groups in adults: *C.leptum*, *C.coccoides*, *Bacteroides* and *Bifidobacterium.* In elderly: increased *E. coli*, decreased beneficial anaerobes like *Bacteroides* and Bifidobacteria. Firmicutes/Bacteroidetes ratio for infants, adults and elderly: 0.4, 10.9 and 0.6, respectively.
Biagi et al. (2010)	84	21 centenarians (99-104 yo) 43 elderly (63-78 yo) 20 young adults (25-40 yo)	Similar microbiota between young adults and 70- year olds. Differences in centenarians: rearranged *Firmicutes* (lower *Clostridium* cluster XIVa, altered Clostridium cluster IV such as decreased *F.prausnitzii*), increased facultative anaerobes like *Proteobacteria* and *Bacilli*, increased *E.limosum* and *A.muciniphila.*
Claesson et al. (2011)	170	161 subjects> 65 yo 9 young controls	Significant differences in core microbiota between young and elderly; elderly have lower Firmicutes proportion, dominated by *Clostridium* cluster IV (*Ruminococcaceae* family).
Park et al. (2015)	131	Individuals from all age groups from urbanized town communities (UTC) and longevity villages communities (LVC)	Lower Firmicutes/Bacteroidetes ratio in LVC; higher levels of *Bacteroides*, *Prevotella*, *Lachnospira*; unique *Bacteroides* spp. and *Faecalibacterium* spp. in LVC. Fecal lipopolysaccharide (LPS) was higher in UTC than in LVC.
Jeffery et al. (2016)	384	371 subjects aged 64-102 yo 13 younger adults(28-46 yo)	Elderly subjects experienced temporal instability in their gut microbiota, especially those with low initial diversity. Long-term care residents losed health-associated components and gained elderly-associated microbes over time. Community-dwelling subjects’ microbiota was more affected by antibiotics, but showed better recovery.
Odamaki et al. (2016)	367	Subjects 0-104 yo	Actinobacteria decreased significantly after weaning and continued to decline with age. Firmicutes was the dominant phylum post- weaning, but less abundant in children <4 years compared to older subjects. Bacteroidetes and Proteobacteria increased in subjects >70 years. *Proteobacteria* abundance changed inversely to Firmicutes.
Wu et al. (2019)	65	17 young subjects (21-33 yo) 23 elderly (68-88 yo) 19 centenarians (99-107 yo)	Centenarians exhibited a more diverse core microbiota than the young and elderly. The gut microbiota of centenarians had reduced levels of *F.prausnitzii* and *E.rectale*, and increased levels of *Methanobrevibacter smithii* and *Bifidobacterium adolescentis*. It had also a high capacity for glycolysis and fermentation to short- chain fatty acids.
Vaiserman et al. (2020)	1550	153 subjects 0-9 yo 93 subjects 10-19 yo 256 subjects 20-29 yo 451 subjects 30-39 yo 321 subjects 40-49 yo 190 subjects 50-59 yo 67 subjects 60-69 yo 19 subjects 70+ yo	Firmicutes/Bacteroidetes ratio increases from ages 0-9 to 60-69, decreases in advanced age (70+); composition of intestinal flora changes significantly across age groups.

A study comparing fecal microbiota among infants, adults, and the elderly revealed continuous changes with aging (Mariat et al., 2009). In infants, *Bifidobacterium* was the most abundant group. In adults, the dominant bacterial groups included *Clostridium leptum, Clostridium coccoides, Bacteroides*, and *Bifidobacterium*, with sub-dominant groups including Lactobacilli, Enterobacteriaceae, and others. In elderly subjects, there was a significant increase in *Escherichia coli* counts and a noted decrease in beneficial anaerobes like *Bacteroides* and Bifidobacteria. The Firmicutes/Bacteroidetes ratio for infants, adults, and elderly subjects was estimated at 0.4, 10.9, and 0.6, respectively.

Another study in Italy found microbiota similarities between young adults and seventy-year-olds but significant differences in centenarians (individuals surpassing 100 years old) (Biagi et al., 2010). Centenarians displayed a rearrangement in Firmicutes population and an abundance of facultative anaerobes, predominantly from Proteobacteria (including *E. coli, Klebsiella pneumoniae,* and *Pseudomonas*) and Bacilli (such as *Bacillus* and *Staphylococus*). The rearrangement in Firmicutes included lower levels of *Clostridium* cluster XIVa, such as *Roseburia intestinalis* and *Ruminococcus obeum*, higher levels of Bacilli, and alterations in the composition of *Clostridium* cluster IV, such as a decrease in *Faecalibacterium prausnitzii*. This also reflected a decrease in butyrate producers, such as *R. intestinalis, R. obeum*, and *F. prausnitzii*. The mucin-degrading *Akkermansia muciniphila* was found to be enhanced in older people. *Akkermansia muciniphila* is recognized for its ability to break down mucin and enhance intestinal integrity by lowering toxicity levels linked to high-fat diets; thus it has beneficial effects on preventing and improving metabolic disorders and obesity (Badal et al., 2020; Xu et al., 2020). Researchers also noted an increase in *Eubacterium limosum* (*Clostridium* cluster XV) in centenarians, suggesting that it could be an indicator of longevity (Biagi et al., 2010).

Other research involving elderly subjects has demonstrated core microbiota differences compared to young adults, with a lower proportion of Firmicutes and a shift towards *Clostridium* cluster IV, specifically within the Ruminococcaceae family, including *Faecalibacterium, Sporobacter*, and *Ruminococcus* species (Claesson et al., 2011). A study comparing urbanized regions with longevity villages—specific regions where residents tend to live significantly longer compared to global averages—revealed a lower Firmicutes to Bacteroidetes ratio in longevity villages residents, along with higher levels of *Bacteroides, Prevotella*, and *Lachnospira* (Park et al., 2015). Notably, some *Bacteroides* spp. and *Faecalibacterium* spp. were found only in longevity villages residents. In contrast, lipopolysaccharide (LPS) was found higher in urbanized towns residents, a finding that is associated with greater consumption of animal-based foods, reduced vegetable intake, and increased intestinal bacteria. LPS promotes chronic low-grade inflammation, a known contributor to aging and related diseases. Conversely, longevity villages residents exhibited higher levels of anti-inflammatory *Faecalibacterium* spp., which may help reduce LPS production and inflammation.

Another study about the gut microbial composition of residents from longevity villages of South China revealed higher amounts of *Enterococcus, Lactobacillus*, Enterobacteriaceae, *Clostridium perfringens*, and *Bacteroides* compared to a control group. Additionally, some unique species, such as *Methanobacterium, Butyricimonas, Deinococcus*, and members of the Streptococcaceae family, were detected in the villagers’ gut microbiota (Yu et al., 2015).

Differences have also been detected between the gut microbiota of centenarians in rehabilitation hospitals and those living at home. Centenarians residing in rehabilitation hospitals had higher levels of Bacteroidetes and Proteobacteria, lower bacterial diversity, and a lower abundance of *Faecalibacterium* compared to those living in the community (Kim et al., 2019). This contrast indicates the impact of the environment and diet on the gut flora composition.

Moreover, a large-scale study conducted in Ukraine indicated age-related variations in intestinal flora. In particular, the Firmicutes/Bacteroidetes ratio increased from ages 0–9 to 60–69), followed by a decrease in the advanced age group (70+) (Vaiserman et al., 2020). These findings are consistent with previous studies (Mariat et al., 2009).

At both the metagenomic and functional levels, the gut microbiota of centenarians exhibits an abundance of genes involved in glycolysis and SCFA production, despite a limited capacity for carbohydrate degradation and amino acid synthesis (Wu et al., 2019; Badal et al., 2020). This model is supported by a high presence of *Bifidobacterium adolescentis, Methanobrevibacter smithii, Escherichia*, and *Lactobacillus*, along with a lower presence of *F. prausnitzii, Eubacterium hallii, Eubacterium ventriosum,* and *Eubacterium rectale* (Biagi et al., 2010; Wu et al., 2019). SCFAs possess anti-inflammatory action by regulating cytokine secretion and immune cell function by mechanisms like direct action on immune cells and inhibition of nuclear factor kappa B (NF-κB) activity (Liu et al., 2021; Yao et al., 2022). Among them, butyrate has been highlighted for its anticarcinogenic properties, maintaining the intestinal barrier integrity as well as the modulation of oxidative stress by regulating oxidoreductase activity and inhibiting the production of reactive oxygen and nitrogen species (Liu et al., 2021; Visekruna and Luu, 2021).

Additionally, genes involved in menaquinone (vitamin K2) and riboflavin (vitamin B2) biosynthesis are increased in centenarians (Wu et al., 2019). Menaquinone supports bone health, reduces the risk for coronary heart disease, and decreases the overall cancer incidence and mortality (Buchanan, MD et al., 2016), while riboflavin is vital for various redox reactions in human metabolism (Olfat et al., 2022). Decreased trimethylamine (TMA) levels, a metabolite associated with cardiovascular disease, cancer, and metabolic disease, have also been reported among centenarians, possibly mediated by *M. smithii* (Wu et al., 2019; Gatarek and Kaluzna-Czaplinska, 2021). Centenarians also possess a unique gut microbiome enriched with microorganisms capable of producing distinctive secondary bile acids, including various forms of lithocholic acid (LCA) such as iso-, 3-oxo-, allo-, 3-oxoallo-, and isoallolithocholic acid. These are reported to suppress pro-inflammatory T helper 17 cells and induce Treg cells (Sato et al., 2021; Salazar et al., 2023).

## 4 Factors contributing to age-related gut microbiota dysbiosis

Taking into account the influence of diet on gut microbiota composition, alterations in the nutritional habits and lifestyles of older individuals contribute to age-related imbalances in the intestinal microbial community. The elderly experience a decreased chewing ability, tooth loss, and diminished taste perception, factors affecting appetite and dietary choices, that can ultimately impact their gut microbiota composition (Claesson et al., 2011). In particular, they prefer foods richer in sugar and fat while reducing the consumption of plant-based foods. However, a balanced diet, rich in micronutrients and low in saturated fats, is crucial for longevity, as evidenced by populations in regions with high life expectancies (Kahleova et al., 2021). Centenarians in longevity villages are reported to maintain regular and diverse dietary habits, typically eating three meals a day with appetite. Their intake of protein and other nutrients is similar to that of adults in the same region (Kim et al., 2019).

Furthermore, the elderly experience reduced intestinal function compared to younger individuals, which may lead to constipation and impact digestion, nutrient absorption, and immune activity (Woodmansey et al., 2004; Claesson et al., 2012; Odamaki et al., 2016). Moreover, shifts in body mass index (BMI) during aging may be reflected in the observed microbiota changes. An increased BMI is associated with a higher proportion of Firmicutes and a lower proportion of Bacteroidetes (Koliada et al., 2017).

**FIGURE 2:**
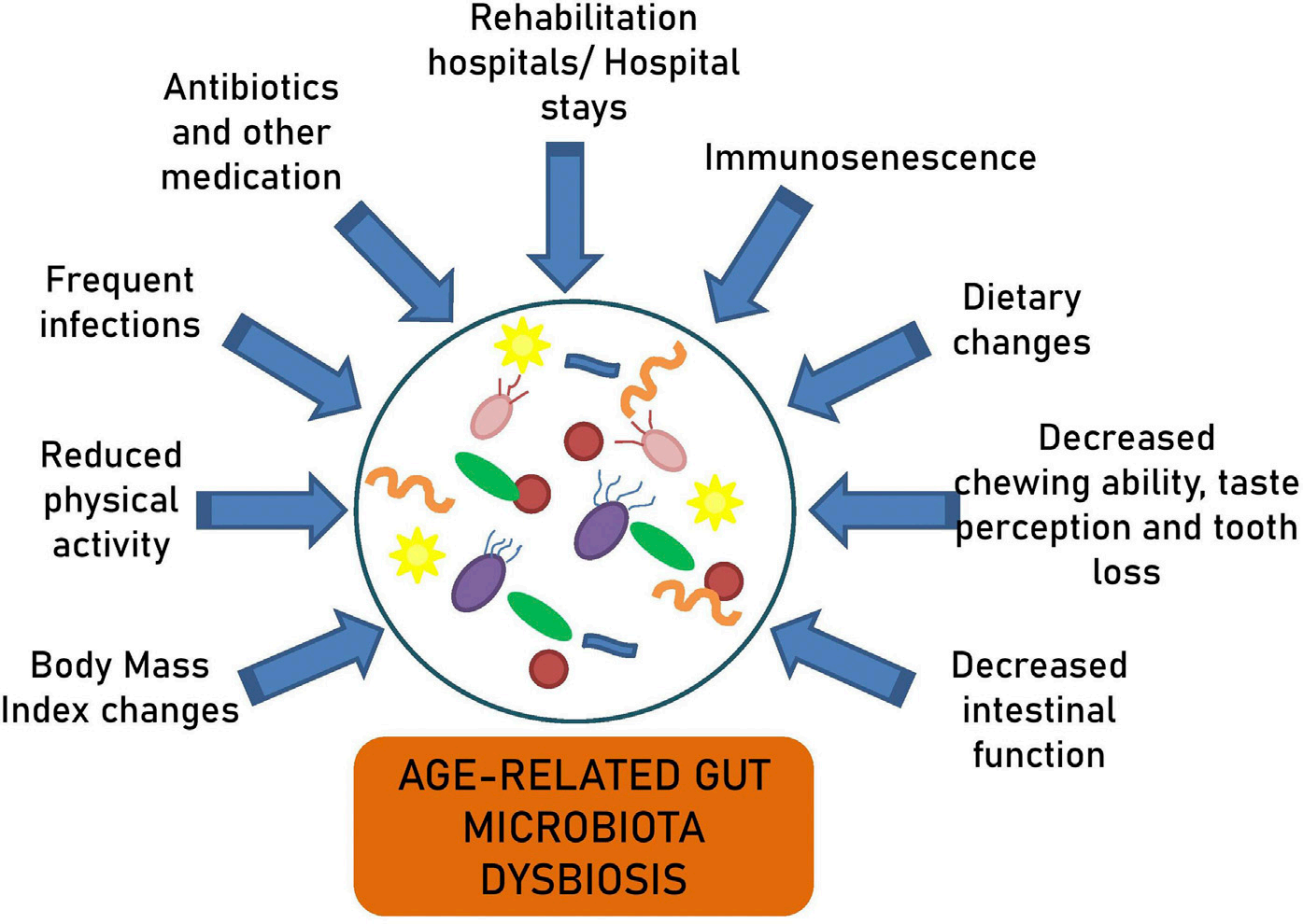
Factors contributing to age-related gut microbiota dysbiosis.

Multiple other factors explain the age-related microbiota alterations, such as shifts in immune response, hospital stays, extended intestinal transit times, decreased physical activity, recurrent infections, and the frequent use of antibiotics and other medications (Figure 2) (Mariat et al., 2009; Claesson et al., 2012; Lakshminarayanan et al., 2014; Odamaki et al., 2016). It has been reported that long-term care residents lose their health-associated components and gain elderly-associated microbes over time (Jeffery et al., 2016). The comparison of the gut microbiota between centenarians living in the community and those in rehabilitation hospitals has shown higher proportions of Bacteroidetes and Proteobacteria, a lower proportion of *Faecalibacterium,* and lower bacterial diversity in the latter (Kim et al., 2019). Similarly, long-stay residents exhibited a decrease in *Ruminococcus* and *Prevotella* and an increase in *Oscillibacter* compared to community-dwelling subjects (Claesson et al., 2012). These microbiota alterations were associated with the composition and variety of their diets.

## 5 Inflammaging, immunosenescence and the gut microbiota

The gut microbiota significantly impacts the health of older individuals by contributing to immune system development, protection against pathogens, and maintenance of intestinal barrier integrity (Claesson et al., 2012). It induces the production of antimicrobial peptides, mucins, and IgA immunoglobulins through dendritic cell activation, in response to microbial molecules recognized by pattern recognition receptors (Gyriki et al., 2024). IgA, which is essential for gut balance, is produced by the gut-associated lymphoid tissue (GALT) following microbiota colonization (Yoo et al., 2020). Research involving germ-free animals has shown that the microbiota aids in the formation of GALT and enhances the mucosal barrier (Min and Rhee, 2015). Microbial metabolites, like butyrate and tryptophan, enhance interleukin-22 (IL-22) production by innate lymphoid cells, contributing to gut homeostasis (Min and Rhee, 2015; Shi et al., 2017). Additionally, the gut microbiota contributes to the development of T helper 17 (Th17) and regulatory T cells (Treg) in the colon, which are necessary for controlling the proliferation of CD4^+^ Th cells in response to commensal bacteria (Min and Rhee, 2015).

Studies on centenarians, often associated with healthy aging, reveal a higher abundance of genes involved in SCFA production, which possess anti-inflammatory properties and protect the gut barrier (Wu et al., 2019; Liu et al., 2021). SCFAs directly influence immune responses by stimulating immune cells, promoting neutrophil recruitment, inhibiting NF-κB and tumor necrosis factor alpha (TNFα), supporting B cell differentiation, and regulating systemic immune responses to protect against allergic diseases and neuroinflammation (Ragonnaud and Biragyn, 2021). Butyrate, a key SCFA, enhances memory responses in CD8^+^ T cells and promotes anti-inflammatory responses by inducing Tregs and IL-10-producing T cells, while inhibiting pro-inflammatory cytokines (IFN-γ, IL-6, IL-1β) (Ragonnaud and Biragyn, 2021). Secondary bile acids, such as lithocholic acid, represent another vital metabolic by-product of the gut microbiota that regulates immune responses by inhibiting Th-17 cells and promoting Treg cells (Sato et al., 2021; Salazar et al., 2023). *Akkermansia muciniphila*, enriched in older age, enhances colonic mucus production, supports the growth of commensal bacteria, and mitigates immune system processes by reducing CD80 and CD273 B cell activation in Peyer’s patches (Ragonnaud and Biragyn, 2021; Salazar et al., 2023).

Advanced age is linked to chronic low-grade inflammation, termed “inflammaging,” which is associated with systemic and local pathologies, like neuroinflammation and arteriosclerosis (López-Otín et al., 2023). The age-related rise in circulating inflammatory mediators and biomarkers, such as cytokines and C-reactive protein (CRP), is a recognized phenomenon, yet the root cause remains a topic of debate (Thevaranjan et al., 2017; López-Otín et al., 2023). Remarkably, anti-inflammatory treatments, such as anti-TNFα agents, have shown pro-longevity effects and restoration of age-related pathological conditions in mouse studies (Sciorati et al., 2020; Minhas et al., 2021).

Inflammaging is closely linked to immunosenescence, which describes the marked changes and deterioration of the immune system with age (Fulop et al., 2018). The balance between pro-inflammatory and regulatory responses is disrupted, leading to a state of low-grade chronic systemic inflammation. Immunosenescence starts around 50 years of age and makes individuals more prone to infections, autoimmunity, cancer, and less effective vaccination (Huang et al., 2022). Key features include a decrease in naïve T and B cells alongside an increase in memory cells, impaired function of NK cells, and senescent macrophages secreting pro-inflammatory cytokines (Fulop et al., 2018; Huang et al., 2022). Changes in T cell populations lead to increased activity of pro-inflammatory Th1 and Th17 cells, impaired immunosurveillance, increased autoimmune diseases, and weakened biological barriers, all of which contribute to systemic inflammation (López-Otín et al., 2023).

Centenarians appear to manage chronic inflammation by balancing pro- and anti-inflammatory responses, a phenomenon known as “anti-inflammaging” (Antuña et al., 2022). Specifically, they counteract pro-inflammatory cytokines by harmonizing them with anti-inflammatory signals, achieved by reducing the ratio of Th17 and regulatory T cells (Th17/Treg) and shifting their secretory profile toward anti-inflammatory phenotypes. Such balance, together with modifications in the activity of immune cells and autophagy, can prevent age-related conditions like sarcopenia. Immunomodulation has been suggested as a promising therapeutic option for sarcopenia prevention. Physical exercise, biophysical stimulation, nutrition, and drug therapies have been carried out to target immune cells, myokines, autophagy, and the gut microbiota, thus performing an immunomodulatory action, with possible applications in sarcopenia management (Zhang et al., 2024).

There is a well-established link between inflammaging and dysbiosis. The altered gut microbiota of older individuals increases intestinal permeability and activates the immune system, resulting in chronic inflammation, frailty, and morbidity (Claesson et al., 2012; López-Otín et al., 2023). In *Drosophila*, elevated Gammaproteobacteria in older age have been linked to increased intestinal permeability and immune activation (Clark et al., 2015). In humans, elevated pro-inflammatory IL-6 and IL-8 in centenarians correlate with an increase in Proteobacteria and a decrease in butyrate-producing bacteria such as *Eubacterium rectale, Eubacterium hallii*, and *Eubacterium ventriosum* (Biagi et al., 2010). Notably, despite a decrease in many anti-inflammatory Firmicutes species with age, there is a significant increase in *Eubacterium limosum*, which may have beneficial anti-inflammatory properties (Biagi et al., 2010). This species can convert dietary isoflavonoids into phytoestrogens—potentially protective against cancer and coronary heart disease—and primary bile acids (taurochenodeoxycholic acid and glycochenodeoxycholic) into anti-inflammatory secondary bile acids, such as ursodeoxycholic acid (UDCA) and lithocholic acid (LCA) (Huang, 2023; Zhou et al., 2023).

In a study, transferring gut microbiota from young or old conventional mice to young germ-free mice concluded that aged microbiota is related to chronic low-grade inflammation that characterizes aging (Fransen et al., 2017). The microbiota from older mice increased Th2, Th1, and splenic Treg cell frequencies, inflammatory markers like TNF-α in their ileum, and altered gut microbiota composition—increased TM7 and Proteobacteria, both linked to intestinal inflammation, and decreased *Akkermansia*. This transfer may increase the translocation of inflammatory bacterial components into the bloodstream due to impaired gut barrier.

Similarly, the study by Thevaranjan et al. utilized both young and old germ-free and conventional mice to illustrate that age-related microbiota alterations play a significant role in increasing intestinal permeability, inflammation linked with age, and declining macrophage function (Thevaranjan et al., 2017). They demonstrated that aging promotes gut leakiness, allowing microbial components to enter the circulation and trigger systemic inflammation, indicated by high levels of serum IL-6 and TNF. Importantly, lowering TNF levels counteracts microbiota changes and shields older mice from increased intestinal permeability.

## 6 Microbiota and age-related diseases

The process of aging can disrupt the body’s internal equilibrium and hormonal regulation, potentially resulting in metabolic conditions like diabetes and obesity (Khaledi et al., 2024). Changes in the microbiota are believed to contribute to various pathologies, including frailty, neurodegenerative diseases, insulin resistance, type 2 diabetes, cancer, and cardiovascular disease (Bischoff, 2016; Ragonnaud and Biragyn, 2021). Despite a surge in reports linking gut microbiota to health during aging, the field remains poorly understood and appears confusing. Table 2 summarizes the possible mechanisms linking the microbiota to aging, including inflammaging, altered microbial production, neuroinflammation, immune dysregulation, and induction of cellular stress.

**TABLE 2:** Mechanisms Linking Microbiota and Aging.

Mechanism	Description/Pathways Involved	Key Microbial Factors/Metabolites	Impact on Aging
Inflammaging	Dysbiosis increases intestinal permeability, allowing bacterial components (e.g., LPS) to translocate into circulation. This triggers chronic, low- grade systemic inflammation.	LPS, elevated pro- inflammatory cytokines (e.g., TNF-α, IL-6)	Promotes frailty, accelerates age-related systemic inflammation, and contributes to tissue damage.
Altered Metabolite Production	Changes in the composition of the microbiota lead to an imbalance in microbial metabolites. A decline in beneficial molecules such as SCFAs reduces gut barrier integrity.	Reduced butyrate and other SCFAs; altered secondary bile acids (e.g., conversion to UDCA and LCA), elevated TMAO	Impairs gut barrier function and metabolic homeostasis, thereby exacerbating aging- related declines. TMAO increases risk of cardiovascular events and may contribute to type 2 diabetes mellitus pathogenesis.
Neuroinflammation and BBB Disruption	SCFAs can cross the BBB, influence microglia activity, and reduce inflammation. Microbial dysbiosis can increase gut permeability and introduce bacterial products like muramyl dipeptide and LPS into the bloodstream, leading to increased BBB leakage.	Decreased SCFA levels (e.g., butyrate), increased bacterial products like LPS and muramyl dipeptide	Contributes to neurodegenerative changes seen in Alzheimer’s disease, Parkinson’s disease, and cognitive decline.
Immune Dysregulation	Altered gut microbiota composition influences immune cell differentiation (e.g., altered Th17/Treg balance) and function, leading to a state of immunosenescence.	Shifts in commensal bacteria; changes in pro- and anti- inflammatory cytokine profiles	Increases susceptibility to infection, autoimmunity, and decreases overall immune competence with age.
Induction of Cellular Stress	Bacterial toxins and metabolic imbalances increase oxidative stress and the activation of cellular senescence pathways.	Microbial toxins (e.g., colibactin from certain *Escherichia coli* strains, CagA and VacA from *Helicobacter pylori*)	Promotes cellular aging, genomic instability, and may enhance the risk for age-associated diseases, especially cancer.

This table summarizes the possible mechanisms linking the microbiota and aging, including inflammaging, altered microbial production, neuroinflammation, immune dysregulation, and induction of cellular stress. LPS: Lipopolysaccharide, TNF-α: Tumor Necrosis Factor-alpha, IL-6: Interleukin-6, SCFAs: Short-Chain Fatty Acids, UDCA: Ursodeoxycholic Acid, LCA: Lithocholic Acid, TMAO: Trimethylamine N-oxide, BBB: Blood-Brain Barrier, Th17: T Helper 17 cells, Treg: Regulatory T cells.

### 6.1 Neurodegenerative diseases

Aging is a significant risk factor for neurodegeneration, with Alzheimer’s disease (AD) and Parkinson’s disease (PD) predominantly affecting older adults (Hou et al., 2019). Brain aging is marked by reduced neuronal activity, irregular glial cell activation, stem cell depletion, and disrupted metabolism, which can lead to cognitive decline and neurodegenerative diseases (Mattson and Arumugam, 2018).

Microglia, comprising 10%–15% of brain cells, are key immune defenders in the central nervous system (CNS), and altered microglial morphology and function can lead to brain inflammation and cognitive decline (Saitgareeva et al., 2020; Zhou et al., 2022). A diverse gut microbiota, primarily through the action of SCFAs, is essential for normal microglia maturation (Erny and Prinz, 2020). The vagus nerve, which links the gut and the brain, plays a role in the interaction between the microbiota and microglia (Zhou et al., 2022). The blood-brain barrier (BBB) typically protects the CNS from pathogens, but its integrity diminishes with age (Hussain et al., 2021). Dysbiosis in the elderly may increase gut permeability and introduce bacterial products like muramyl dipeptide and LPS into circulation, leading to increased BBB leakage (Thevaranjan et al., 2017). SCFAs can cross the BBB, influence microglia activity, and reduce inflammation; notably, butyrate can restore BBB permeability (Erny and Prinz, 2020).

#### 6.1.1 Alzheimer’s disease

Alzheimer’s disease (AD), the leading cause of dementia in individuals over 60, is characterized by neurodegeneration, neuronal loss, and the formation of neurofibrillary tangles and amyloid-beta (Aβ) plaques (Srivastava et al., 2021).

The gut microbiota of AD patients shows notable changes, suggesting a potential link between the gut microbiota and the AD pathogenesis. AD patients exhibit reduced microbial diversity and significant differences at various taxonomic levels, including decreased Firmicutes and *Bifidobacterium* and increased Bacteroidetes (Vogt et al., 2017). Also, a higher abundance of the pro-inflammatory taxa *Escherichia/Shigella* and a lower abundance of the anti-inflammatory taxon *E. rectale* have been detected in patients with cognitive decline and brain amyloidosis, which is linked to peripheral inflammation (Cattaneo et al., 2017). These microbial changes are thought to contribute to a pro-inflammatory environment through mechanisms such as increased intestinal permeability, which may lead to systemic inflammation and impact neuroinflammatory processes in the brain.

Animal models support this link; for instance, APP/PS1 transgenic mice, a model for AD, exhibit elevated levels of Helicobacteraceae and Desulfovibrionaceae, as well as *Odoribacter* and *Helicobacter* genera, along with a reduction in *Prevotella* compared to wild-type mice (Shen et al., 2017). Other studies have noted a decreased presence of *Bacteroides* and an increased abundance of *Ruminococcus* species in AD patients (Zhuang et al., 2018). Together, these findings suggest that gut dysbiosis might influence AD development and progression, possibly by promoting an inflammatory milieu that exacerbates neurodegeneration.

Microglia play a crucial role in clearing Aβ plaques and maintaining cerebral balance. However, prolonged exposure to Aβ can activate microglia, resulting in chronic neuroinflammation and neurotoxicity. As the interplay between microglia and microbiota is already mentioned, the gut microbiota could influence the AD pathogenesis.

Additionally, the secretion of microbial metabolites by the gut flora is implicated in AD pathogenesis. *Bacteroides fragilis*-derived lipopolysaccharides (BF-LPS) are recognized by microglial receptors TLR2, TLR4, and/or CD14, similar to pro-inflammatory amyloid-beta (Aβ42) peptides found in AD brains (Lukiw, 2016). Elevated levels of *Escherichia coli* K99 and LPS have been observed in AD brains, with LPS colocalized with Aβ1-40/42 in amyloid plaques and around blood vessels (Zhan et al., 2016). This suggests a gut-to-brain connection, with the gut potentially serving as a source of brain *E. coli*. Furthermore, higher bacterial LPS have been found in brain samples from the hippocampus and superior temporal lobe neocortex of AD patients compared to age-matched controls (Zhao et al., 2017). These findings indicate that the gut microbiome, particularly Gram-negative bacilli like *Bacteroides fragilis* and *E. coli*, produce neurotoxic substances like LPS that can cross physiological barriers to reach the CNS. Age-related changes in the integrity of the gut and BBB, leading to increased leakiness, are implicated in promoting systemic inflammation, neuroinflammation, and neurodegeneration.

#### 6.1.2 Parkinson’s disease

Parkinson’s disease (PD) is the second most prevalent progressive neurodegenerative disorder, affecting about 2%–3% of 65-year-olds and older (Poewe et al., 2017). It is characterized by motor symptoms like resting tremor and bradykinesia, as well as non-motor symptoms like gastrointestinal dysfunction and constipation. The pathophysiology of PD involves the loss of dopaminergic neurons in the substantia nigra and the accumulation of α-synuclein within neuronal cells.

Current research suggests that changes in the gut microbiota play a role in PD pathogenesis. In a PD murine model, fecal analysis exhibited a decrease of Firmicutes and Clostridiales, along with an increase of Proteobacteria, Turicibacterales, and Enterobacteriales, and higher fecal SCFA levels (Sun et al., 2018). Moreover, studies demonstrated differences in colonic and fecal microbiota in PD subjects compared to healthy controls; anti-inflammatory bacteria like *Faecalibacterium, Blautia*, and *Coprococcus* were less abundant in PD patients, while pro-inflammatory bacteria like Proteobacteria of the genus *Ralstonia* were more prevalent (Keshavarzian et al., 2015). Notably, genes related to the LPS production and type III bacterial secretion systems were highly abundant in PD subjects. Bacteria from the Prevotellaceae family were also decreased in the gut microbiota of PD patients (Scheperjans et al., 2015). In another study, PD patients exhibited a distinct microbiota composition with enriched levels of Verrucomicrobia, *Mucispirillum, Porphyromonas, Lactobacillus*, and *Parabacteroides*, and depleted *Prevotella*. Additionally, *Bacteroides* were more abundant in non-tremor PD patients compared to those displaying the tremor subtype (Lin et al., 2019). The observed microbiota changes were linked to altered plasma levels of IFN-γ and TNF-α.

Summarizing, dysbiosis is a feature found in PD and may contribute to neuroinflammation and disease pathogenesis. Pro-inflammatory gut dysbiosis may lead to inflammation-induced misfolding of α-synuclein (Keshavarzian et al., 2015). The TLR4/TBK1/NF-κB/TNF-α signaling pathway can be a key factor in PD, since the activation of TLR4 by LPS-containing bacteria triggers gut inflammation, which in turn activates microglia as well as neuroinflammation (Sun et al., 2018). SCFAs may also promote the overactivation of microglia and astrocytes in PD by crossing the BBB and affecting the physiology of brain cells, including microglia maturation (Sun et al., 2018). A recent cohort study suggested that plasma levels of the SCFAs were higher in PD patients, while fecal levels of SCFAs were lower, correlating with alterations in gut microbiota and the clinical severity of PD (Chen et al., 2022). Findings are in accordance with previous research that also reported elevated plasma levels of SCFA in PD patients (Shin et al., 2020).

### 6.2 Diabetes mellitus type 2

Type 2 diabetes mellitus (T2DM) is a metabolic disorder of age that imposes a significant global health burden. Increasing evidence suggests that the gut microbiota is involved in the development and progress of T2DM.

One study of male C57 BL/KS db/db mice, a model for T2DM, revealed significant alterations in the gut bacterial diversity and abundance. These included increased levels of Verrucomicrobia and family S247 and decreased levels of Bacteroidaceae and Prevotellaceae in db/db mice. Transplantation of fecal bacteria from db/db and m/m mice into pseudo-germfree mice induced notable changes in metabolic parameters, including body weight, fasting blood glucose, fluid and food intake, and composition of gut microbiota. The findings suggest that disturbances in gut microbiota may contribute to T2DM development and that therapeutic interventions targeting the microbiota could benefit individuals with T2DM and age-related glucose intolerance (Yu et al., 2019).

In patients with T2DM, a higher abundance of the *Collinsella* genus and an unidentified genus within the Enterobacteriaceae family, along with dysbiosis, have been described (Lambeth et al., 2015). Additionally, there is a decline in the prevalence of some universally present butyrate-producing bacteria and a rise in diverse opportunistic pathogens, with enhanced microbial functions associated with sulfate reduction and resistance to oxidative stress (Qin et al., 2012). Certain genera, including *Bifidobacterium, Bacteroides, Faecalibacterium, Akkermansia*, and *Roseburia,* are inversely correlated with T2D, whereas *Ruminococcus, Fusobacterium*, and *Blautia* are positively correlated (Gurung et al., 2020). Prediabetic individuals also present with gut microbiota alterations involving reduced counts of butyrate-producing bacteria, like *A. muciniphila* and *Faecalibacterium prausnitzii*, along with lower abundance of Verrucomicrobiae, suggesting an indicator of T2DM (Zhang et al., 2013).

Microbial metabolic byproducts like SCFAs, tryptophan metabolites, TMAO, LPS, and BAs may be crucial contributors to T2DM pathogenesis (Wu et al., 2023). For instance, propionate has been shown to enhance the expression of peptide YY (PYY) and glucagon-like peptide-1 (GLP-1), resulting in weight loss and glycemic control in animal studies (Zhang et al., 2022). Tryptophan metabolites can reduce fat accumulation, blood glucose levels, appetite, and control inflammation (Wu et al., 2023). In a case-control study, plasma TMAO levels were notably higher in diabetes patients compared to healthy individuals (Kalagi et al., 2022). The role of LPS in inducing systemic inflammation and contributing to metabolic diseases like T2DM has already been highlighted. Secondary BAs produced by gut microbes can also stimulate the production of GLP-1, further impacting metabolic regulation (Gurung et al., 2020).

There is compelling evidence that T2DM treatments can extend lifespan and improve health by altering the gut microbiota. Metformin, a commonly used diabetes medication, has been suggested to potentially slow down the aging process in animal and human models (Campbell et al., 2017; Sunjaya and Sunjaya, 2021; Cheng et al., 2022). Studies in *Caenorhabditis elegans* have shown that metformin can extend lifespan, possibly by affecting metabolism and lessening the risk and severity of various diabetes-related conditions, such as cardiovascular diseases, cancer, and neurodegenerative disorders (Mohammed et al., 2021). Meta-analyses have concluded that T2DM patients using metformin experience significantly lower overall mortality, not only compared to other diabetics on other treatments like insulin and sulfonylurea, but even to non-diabetic individuals (Campbell et al., 2017).

Recent studies suggest that metformin may prolong lifespan by modifying the gut microbiome, particularly through alterations in microbial folate and methionine metabolism (Cabreiro et al., 2013). It also alters the gut microbiota by promoting the growth of bacteria that produce SCFAs and enhance the intestinal barrier. This leads to higher circulating SCFA levels and lower levels of immune-activating microbial products (such as LPS, flagellin, and bacterial nucleic acids), which together help shift the balance away from inflammaging and support healthy aging (Prattichizzo et al., 2018). Another potential mechanism by which metformin may extend lifespan through the microbiota is by reducing gut permeability and inflammation. Metformin treatment in older mice increased mucin production in the colon by promoting the growth and differentiation of goblet cells (Ahmadi et al., 2020). This effect, linked to the suppression of Wnt signaling, suggests that metformin helps reduce gut leakiness and inflammation by enhancing the formation of goblet cells and their mucin secretion.

Evidence suggests that, in addition to metformin, other T2DM treatments can slow aging by modulating the gut microbiota. Acarbose has been found to extend lifespan in mice by improving glucose metabolism, enhancing gut health, and increasing levels of beneficial SCFAs—effects achieved by changing the composition and function of the gut microbiota, similar to calorie restriction (Wu et al., 2022). Similarly, in a randomized clinical trial involving T2DM patients with cardiovascular risk factors, a three-month treatment with empagliflozin altered the gut microbiota by increasing beneficial SCFA-producing bacteria (such as *Roseburia, Eubacterium,* and *Faecalibacterium*) and reducing harmful bacteria (such as *Escherichia-Shigella, Bilophila,* and *Hungatella*) (Deng et al., 2022).

### 6.3 Cardiovascular disease

Atherosclerosis, a primary culprit of heart disease and stroke, accounts for roughly 50% of all cardiovascular fatalities. The conventional risk factors linked to atherosclerosis encompass hypertension, hyperlipidemia, diabetes, obesity, and smoking (Shen et al., 2021). Recent studies have spotlighted gut dysbiosis as a pivotal element in the development of cardiovascular diseases (CVDs) (Jonsson and Bäckhed, 2017; Mantziaris and Kolios, 2019; Sanchez-Rodriguez et al., 2020; Verhaar et al., 2020; Witkowski et al., 2020; Shen et al., 2021; Vourakis et al., 2021).

Low abundance of Bacteroidetes and intestinal dysbiosis involving opportunistic pathogens like *Enterobacter, Collinsella, Desulfovibrio*, and *Klebsiella* have been linked to the development of atherogenesis and atherosclerotic plaques (Mantziaris and Kolios, 2019). Stool sample analysis from patients with coronary artery disease has revealed enrichment of *Escherichia-Shigella* and *Enterococcus*, along with a decrease of *Faecalibacterium, Subdoligranulum, Roseburia*, and *E. rectale* (Zhu et al., 2018).

Furthermore, a connection between hypertension and altered gut microbiota has been observed in rat models and humans. In hypertension animal models, reduced microbial diversity, notable increases in the Firmicutes/Bacteroidetes ratio, reductions in bacteria producing acetate and butyrate, and an increase in lactate-producing bacteria have been observed (Yang et al., 2015). Similar microbiota dysbiosis has been confirmed in a limited group of human hypertensive patients (Yang et al., 2015).

Heart failure (HF) patients exhibit a unique microbial pattern characterized by a significant decrease in the genera *Blautia* and *Collinsella*, along with two unidentified genera from the families Erysipelotrichaceae and Ruminococcaceae (Luedde et al., 2017). Additionally, a reduction in the butyrate-producing *Faecalibacterium* has been observed in HF patients (Luedde et al., 2017), whereas potentially pathogenic bacteria, including *Campylobacter, Shigella, Salmonella, Yersinia enterocolitica,* and *Candida* species, have been enriched (Pasini et al., 2016).

Researchers have recognized three substances derived from dietary lipid phosphatidylcholine—choline, trimethylamine N-oxide (TMAO), and betaine—that forecast the likelihood of CVD (Wang et al., 2011). Notably, the formation of TMAO involves the metabolism of these dietary components by the gut microbiota. Specifically, intestinal bacteria convert choline and other precursors into trimethylamine (TMA), which is then oxidized in the liver to form TMAO. Elevated plasma levels of TMAO have been associated with an increased risk of major adverse cardiovascular events, highlighting the significant role of gut microbiota in cardiovascular health (Tang et al., 2013). Additionally, TMAO-producing gut bacteria contribute to heightened platelet reactivity and thrombosis risk (Zhu et al., 2016). Another mechanism involves the stimulation of hepatic von Willebrand factor (VWF) synthesis through TLR2 by the gut microbiota, leading to increased VWF plasma levels and arterial thrombosis (Jäckel et al., 2017; Reinhardt, 2019).

An animal model demonstrated that SCFAs derived from the gut microbiota help regulate blood pressure via Olfr78, an olfactory receptor, and G protein-coupled receptor 41, suggesting another connection of the gut microbiota to CVD (Pluznick et al., 2013). Interestingly, propionate, a SCFA known to cause vasodilation in *ex vivo* studies, triggered a rapid decrease in blood pressure in wild-type mice (Pluznick et al., 2013). LPS has also been linked with atherosclerosis and has been shown to impact the stability of atherosclerotic plaques (Jaw et al., 2016; Sieve et al., 2018; Yoshida et al., 2018). It has been reported that levels of *Bacteroides vulgatus* and *Bacteroides dorei* are reduced in the gut flora of coronary artery disease patients. The administration of live *Bacteroides vulgatus* and *Bacteroides dorei* lowered both fecal and plasma LPS levels and was protective against atherosclerosis in mice (Yoshida et al., 2018).

### 6.4 Cancer

Gastric cancer ranks as the fifth most common cancer worldwide and third in cancer-related mortality. *Helicobacter pylori* infection has been recognized as a significant factor for gastric cancer (Smyth et al., 2020). A 10-year follow-up study demonstrated that eradicating *H. pylori* can substantially reduce gastric mucosal inflammation, slow the progression of intestinal metaplasia and atrophic gastritis, and lower gastric cancer risk, especially when performed early (Zhou et al., 2014). *Helicobacter pylori* may contribute to cancer development through the secretion of harmful substances like CagA and VacA that induce stress in the endoplasmic reticulum, trigger autophagy, and oxidative stress in the stomach epithelium (Díaz et al., 2018; Matson et al., 2021).

Colorectal cancer (CRC) ranks as the third most prevalent cancer globally and second in cancer-related fatalities (Baidoun et al., 2021). Individuals with CRC display notably diminished temporal stability in their gut microbiota and increased diversity in *Clostridium leptum* and *Clostridium coccoides* subgroups (Scanlan et al., 2008). *Fusobacterium nucleatum*, along with specific strains of *Bacteroides fragilis,* has been connected to CRC progression through activation of β-catenin signaling and promotion of inflammation (Matson et al., 2021). *Campylobacter jejuni* may also aid tumor development by DNA damage through cytolethal distending toxin production (He et al., 2019). Another bacterial mechanism involves colibactin, a genotoxic metabolite produced by certain Proteobacteria like *E. coli*, which activates senescence-associated secretory phenotype in cancerous or pre-cancerous epithelial cells (Dalmasso et al., 2014).

The gut microbiota has also been linked to pancreatic cancer (PC), which is one of the most fatal cancers in developed countries. A study of Israeli patients with pancreatic adenocarcinoma detected a varied composition of the gut microbiome, with a higher Bacteroidetes to Firmicutes ratio, a higher proportion of Veillonellaceae*, Akkermansia*, and *Odoribacter,* and a lower abundance of health-associated microbial families like Clostridiaceae, Lachnospiraceae, and Ruminococcaceae (Half et al., 2019). Another similar study in the Chinese population demonstrated higher abundance of potentially harmful bacteria like *Veillonella, Klebsiella*, and *Selenomonas* in PC patients, as well as LPS-producing bacteria like *Prevotella, Hallella*, and *Enterobacter,* while beneficial bacteria like *Bifidobacterium* and SCFA-producing bacteria like *Coprococcus, Clostridium IV, Blautia, Flavonifractor*, and *Anaerostipes* were found in lower abundance (Ren et al., 2017). Although significant differences exist in the gut microbial profiles of pancreatic carcinoma patients compared to healthy individuals, it remains unclear whether these alterations are a cause or a consequence of pancreatic cancer; further longitudinal studies are needed to determine if the observed dysbiosis contributes to the development of the disease or results from it.

The transportation of gut microbiota’s metabolites and antigens, such as LPS and bile acids, to the liver through the hepatic portal vein may link gut microbiota to liver cancer (Matson et al., 2021). Although a particular pattern of gut microbiota in hepatocellular carcinoma (HCC) has not been established yet, some general findings include higher levels of *Parabacteroides, Clostridium*, and *Gemmiger* genera, and a lower abundance of *Bifidobacterium* and *Lactobacillus* in HCC patients compared to healthy controls (Rajapakse et al., 2023).

## 7 Potential microbiota-targeting interventions

**FIGURE 3:**
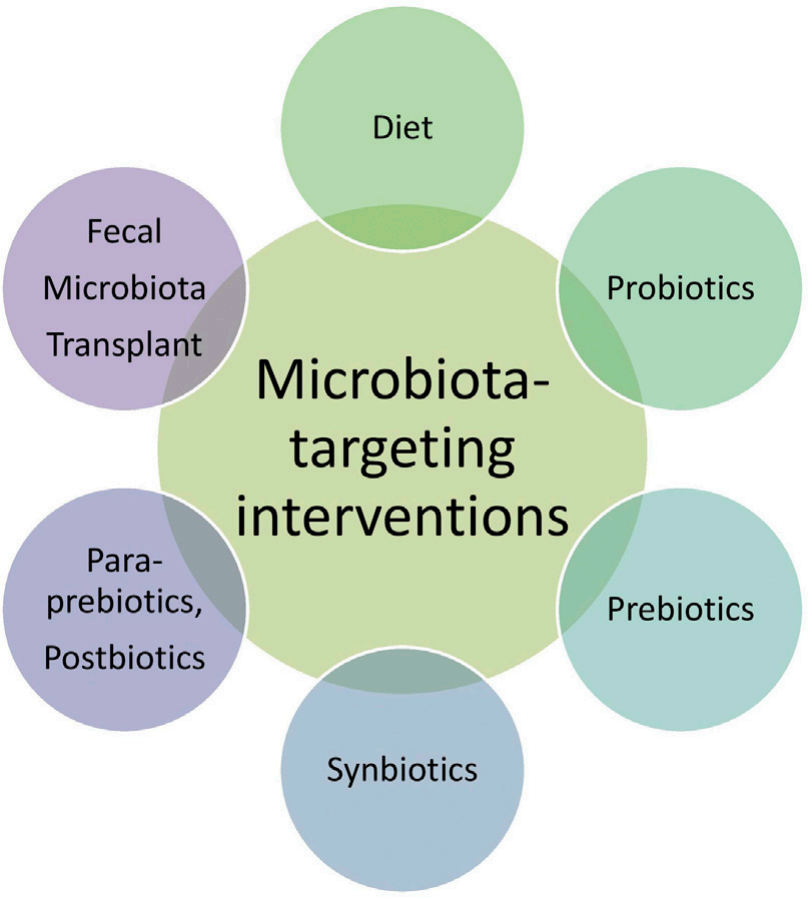
Gut microbiota-targeting interventions for age-related dysbiosis.

Given the critical role of the gut microbiota in aging and age-related diseases, there is a growing interest in microbiota-targeting interventions to promote healthy aging. These interventions include dietary modifications, probiotics, prebiotics, synbiotics, paraprebiotics, postbiotics, and fecal microbiota transplantation (FMT) (Figure 3).

### 7.1 Diet

The well-established link between diet and the intestinal microbiota supports the strategy of using dietary interventions to modulate the microbiota for healthier aging. Dietary supplements could be beneficial in preserving health in older adults by containing specific food ingredients that foster particular components of the microbiota (Claesson et al., 2012). Antibacterial immunity might be enhanced with dietary interventions aimed at decreasing age-related inflammation and macrophage immunosenescence (Thevaranjan et al., 2017). Calorie restriction can lead to compositional alterations in the gut microbiota that contribute to healthy aging, such as increased levels of *Lactobacillus* (López-Otín et al., 2023).

One especially beneficial dietary pattern that has been suggested to have therapeutic effects on frailty is the Mediterranean diet (MedDiet). In a dietary intervention including elderly people from five different countries who received a MedDiet for 1 year, adherence to that diet led to a higher abundance of anti-inflammatory and SCFA-producing bacteria, such as *Faecalibacterium prausnitzii, Roseburia, Eubacterium*, and *Bacteroides thetaiotaomicron*. In contrast, individuals that were not administered the MedDiet had a higher abundance of bacteria associated with age-related diseases, such as *Ruminococcus torques, Collinsella aerofaciens, Coprococcus comes, Dorea formicigenerans, Clostridium ramosum,* and *Veillonella dispar.* These microbial alterations were associated with improved indicators of frailty, enhanced cognitive function, and reduced inflammatory markers, such as C-reactive protein (CRP) and interleukin-17 (IL-17) (Ghosh et al., 2020).

### 7.2 Probiotics

Probiotics and prebiotics might have an impact on delaying or reversing the age-associated microbiota alterations that contribute to morbidities in the elderly (Bischoff, 2016). Probiotics are live microorganisms that confer health benefits when consumed in sufficient amounts, despite not being naturally present in the host’s microbiota. Predominantly comprised of *Lactobacillus* and *Bifidobacterium species*, they also include *Streptococcus thermophilus, Enterococcus faecalis, Enterococcus faecium, Pediococcus,* various Bacilli, and yeasts such as *Saccharomyces boulardii* and *Saccharomyces cerevisiae*, which exhibit similar probiotic properties (Stavropoulou and Bezirtzoglou, 2020).

In a mouse model of accelerated aging, it was shown that the probiotic *Lactobacillus plantarum* GKM3 enhances lifespan and mitigates age-associated cognitive decline (Lin et al., 2021). Replenishing populations of beneficial SCFA-producing bacteria, like *Akkermansia muciniphila,* has also been suggested to improve age-associated conditions (Ragonnaud and Biragyn, 2021; Cani et al., 2022). Reduced levels of *A. muciniphila* have been linked to various diseases in both mice and humans. A study involving oral administration of *A. muciniphila* in mice demonstrated that it significantly alters metabolism by increasing the concentrations of various metabolites with known anti-aging and anticancer properties, such as polyamines (spermidine and spermine), SCFAs like butyrate, and multiple bile acids (Grajeda-Iglesias et al., 2021).

Taking into account the inflammaging and the compromised immune system characterizing the elderly population, the administration of *Bifidobacterium* has been shown to increase lymphocyte levels in the bloodstream, enhance the anti-tumor capabilities of NK cells, and revive the phagocytic function in peripheral blood mononuclear cells and neutrophils (Thevaranjan et al., 2017).

### 7.3 Prebiotics

Prebiotics are non-digestible substances present in food that support the growth of beneficial gut bacteria. They serve as nourishment for the intestinal microbiota, and their breakdown yields SCFAs that can affect not only the gastrointestinal system but also distant organs when absorbed into the bloodstream. Fructo-oligosaccharides and galacto-oligosaccharides are two key types of prebiotics renowned for their beneficial effects on human health (Davani-Davari et al., 2019).

A double-blind, placebo-controlled crossover study investigated the effects of a prebiotic galacto-oligosaccharide mixture on the gut microbiota and immune system of elderly individuals. The results showed that the prebiotic notably enhanced beneficial bacteria, particularly Bifidobacteria, that are found in reduced amounts in the elderly, and improved immune function by increasing phagocytosis, NK cell activity, and IL-10 production, while reducing pro-inflammatory cytokines like IL-6 and TNF-α (Vulevic et al., 2008). An increase in fecal Bifidobacteria in elderly individuals was also observed with a 4-week short-chain fructo-oligosaccharides ingestion (Bouhnik et al., 2007). Freeze-dried powdered yacon (FDY), which contains fructo-oligosaccharides and is considered a prebiotic, was used in another clinical trial to study its effects on glucose, lipid metabolism, and intestinal transit in elderly individuals. The results showed that daily FDY significantly reduced serum glucose levels in the elderly but did not affect the other parameters (Scheid et al., 2014). Conversely, one controlled cross-over study found no significant impact of galacto-oligosaccharides supplementation on immune function or oxidative stress (Wilms et al., 2021).

### 7.4 Synbiotics

The pairing of one or more prebiotics with one or more probiotics, creating a symbiotic blend, has been investigated for its potential to alter the gut microbiota through synergistic effects (Gyriki et al., 2024). Administration of a synbiotic, composed of strains of *Bifidobacterium bifidum* and *Bifidobacterium lactis* and a prebiotic based on inulin, has been reported to increase the abundance of fecal Bifidobacteria of healthy elderly subjects (Bartosch et al., 2005). One study examined the effects of *Lactobacillus rhamnosus* GG and its pilus-deficient variant *L. rhamnosus* GG-PB12 with Promitor™ Soluble Corn Fiber (SCF) on microbiota, metabolism, lipid profile, and immunity in elderly individuals (Costabile et al., 2017). The symbiotic composed of *L. rhamnosus* GG and SCF tended to enhance NK cell activity in elderly women and those aged 70–80 and improved cholesterol levels in hypercholesterolemic patients, while the symbiotic composed of *L. rhamnosus* GG-PB12 and SCF increased NK cell activity more than SCF alone. Concluding, the synbiotic *L. rhamnosus* with SCF showed potential for improving immune and microbial health in the elderly. In another randomized, double-blind, placebo-controlled clinical study, a synbiotic formula of *L. plantarum PBS067, Lactobacillus acidophilus PBS066*, and *Lactobacillus reuteri PBS072* with active prebiotics was used in a large sample of elderly patients with metabolic syndrome and reduced the prevalence of metabolic syndrome, improved various cardiovascular risk factors, and decreased markers of insulin resistance (Cicero et al., 2021). However, data from another study indicated that synbiotic supplementation exerted a mild improvement in gut function in healthy elderly individuals, without notable changes in serum inflammatory markers (Valentini Neto et al., 2020).

### 7.5 Paraprobiotics, postbiotics

Paraprobiotics, also referred to as “non-viable probiotics,” “inactivated probiotics,” or “ghost probiotics,” consist of non-living microbial cells that, when taken in adequate amounts, offer benefits to consumers. While probiotics have shown health advantages, non-viable microbial cells may be safer than live probiotics by reducing the risks of microbial translocation, infection, or increased inflammatory responses, especially in people with imbalanced or weakened immune systems (Aguilar-Toalá et al., 2018).

The term “postbiotics,” which can also be referred to as metabiotics, biogenics, or simply metabolites/cell-free supernatants (CFS), encompasses soluble substances produced by live bacteria or released after bacterial breakdown. These substances, including SCFAs, enzymes, peptides, vitamins, organic acids, and various other compounds, confer physiological benefits through their bioactivity. Despite not fully understanding the mechanisms behind postbiotics’ beneficial effects, scientific evidence suggests they possess various functional properties such as antimicrobial, antioxidant, and immunomodulatory effects (Aguilar-Toalá et al., 2018).

Findings from a clinical trial that evaluated whether heat-killed *Lactobacillus gasseri* can influence the immune response in elderly individuals suggest that it can enhance CD8(+) T cell counts and prevent the loss of CD28 expression in these cells among the elderly, potentially boosting their natural defense mechanisms against infections (Miyazawa et al., 2015). Another clinical trial involved 42 participants aged 65 and above from two nursing homes who were randomly assigned to receive either a jelly containing 10 billion heat-killed *Lactobacillus paracasei* or a placebo jelly for 6 weeks (Maruyama et al., 2016). An influenza vaccination was performed 3 weeks after the initiation of the jelly consumption in order to evaluate the immune responses. No significant effects of the non-viable *L. paracasei* on immune parameters in the overall elderly population were reported, with potential benefits only for the oldest participants.

An animal study aimed to examine the effects and mechanisms of the SCFA acetate on AD, showing its anti-neuroinflammatory effects and its potential as an alternative therapeutic strategy for AD (Liu et al., 2020). Another animal study provided evidence that SCFA supplementation benefits the gut-lung axis in old mice by reducing inflammaging, metabolic dysfunction, and age-related inflammatory exacerbation of acute lung injury (Hildebrand et al., 2023).

### 7.6 Fecal microbiota transplantation (FMT)

FMT, which involves transferring the entire gut microbiota from a healthy donor to a recipient, has received growing attention and research as a treatment for dysbiosis. It has been effectively used for refractory *Clostridioides difficile* infection and shows promise in treating inflammatory bowel disease (Boicean et al., 2023; Gyriki et al., 2024).

FMT has been demonstrated to possess neuroprotective effects in a study of Sun et al. (2018). In this study, fecal material administration from PD mice to wild-type mice led to impaired motor function and reduced striatal neurotransmitters, whereas fecal transplantation in PD mice corrected gut microbial imbalances, decreased activation of brain microglia and astrocytes, lowered fecal SCFAs, and improved motor function (Sun et al., 2018). Another study showed that fecal microbiota transplant from wild-type mice to progeroid mice can extend both the lifespan and healthspan of progeroid mice (Bárcena et al., 2019). Of note, only transplantation with *A. muciniphila* yielded benefits (Bárcena et al., 2019). In another animal model, FMT from young or old mice into aged mice showed that age-related changes and cognitive impairments were alleviated in the aged mice that received microbiota from young donors, supporting the potential of targeting the microbiome for a healthier aging (Boehme et al., 2021). In terms of T2DM, a randomized controlled trial demonstrated that repeated FMTs in obese patients with T2DM increased the extent and persistence of microbiota engraftment and, in combination with lifestyle changes, achieved more beneficial alterations in the recipients’ microbiota and improvements in their lipid levels and liver stiffness (Ng et al., 2022).

## 8 Discussion

Aging is associated with notable shifts in the composition and function of the gut microbiota. Research indicates a decrease in microbial diversity and changes in the abundance of specific bacterial groups in older individuals compared to younger counterparts. For instance, there tends to be an increase in *Escherichia coli* and other Proteobacteria and a decrease in beneficial bacteria like *Bacteroides* and *Bifidobacterium*, essential for gut health and overall wellbeing (Woodmansey et al., 2004; Mariat et al., 2009; Biagi et al., 2010; Odamaki et al., 2016). Centenarians, a unique subset of elderly individuals, serve as a fascinating model for studying longevity and investigating gut microbiota alterations that could potentially facilitate healthier aging. Centenarians exhibit a noteworthy trend: an elevation in genera such as *Akkermansia*, which holds potential implications for longevity (Biagi et al., 2010).

These alterations in the gut microbiota are influenced by several factors, including dietary changes, reduced physical activity, increased medication use, and physiological changes in the gastrointestinal tract such as decreased gut motility (Biagi et al., 2010; Claesson et al., 2011; Claesson et al., 2012; Jeffery et al., 2016; Odamaki et al., 2016; Kim et al., 2019). The decline in beneficial bacteria and the proliferation of potentially pathogenic microbes contribute to an imbalanced gut environment, often referred to as dysbiosis. Dysbiosis in the elderly has been associated with various age-related conditions, like inflammaging, cognitive decline, neurodegeneration, insulin resistance, type 2 diabetes mellitus, cardiovascular disease, and cancer (Bischoff, 2016).

Given the critical role of the gut microbiota in aging and age-related diseases, there is a growing interest in microbiota-targeting interventions to promote healthy aging. Diet plays a crucial role in the shaping of the microbiota. High-fiber diets rich in fruits, vegetables, and whole grains, like the Mediterranean diet, can promote the growth of beneficial bacteria and enhance microbial diversity (Filippis et al., 2016; Mitsou et al., 2017; Garcia-Mantrana et al., 2018; Tsigalou et al., 2021). In addition to dietary modifications, probiotics, non-viable probiotics (paraprobiotics), prebiotics, synbiotics, and microbial soluble factors (postbiotics) have garnered significant attention for their potential to modulate the gut microbiota and enhance the health of the elderly. Several studies have shown that probiotic supplementation can improve gut health, enhance immune function, and reduce inflammation in older adults (Thevaranjan et al., 2017; Grajeda-Iglesias et al., 2021; Lin et al., 2021). Prebiotics have also been shown to beneficially alter the gut microbiota composition and improve metabolic health (Bouhnik et al., 2007; Vulevic et al., 2008), as has synbiotics (Bartosch et al., 2005; Costabile et al., 2017; Cicero et al., 2021). Paraprobiotics and postbiotics also attract attention as microbiota-modulating agencies, as they offer a safer profile than live microorganisms (Miyazawa et al., 2015; Liu et al., 2020; Hildebrand et al., 2023). Although still in the experimental stages for age-related conditions, FMT has shown promise in restoring a healthy microbiota and improving metabolic and immune functions in older adults, based on animal studies (Sun et al., 2018; Bárcena et al., 2019; Boehme et al., 2021; Ng et al., 2022).

While microbiota-targeting interventions hold promise, several challenges and questions remain. First of all, much of the research is based on small-scale or short-term studies, often conducted in animal models rather than human subjects. While these animal studies provide valuable insights, the results are not always directly translatable to humans due to species-specific differences in gut microbiota composition and physiology. For instance, the gut microbiota of mice, commonly used in these studies, differs significantly from that of humans in both composition and function (Nguyen et al., 2015). Moreover, even in human studies, there is interindividual variability in gut microbiota composition, complicating the generalization of findings. Factors such as genetics, diet, lifestyle, medication use, and environmental exposures all contribute to these differences. Also, the variability in study designs and methodologies poses another challenge. For example, populations, sample sizes, definitions of older individuals, intervention strategies, dosages of the treatment, microbiota analysis methods (e.g., 16S rRNA sequencing, next-generation sequencing, and microbiome shotgun sequencing), metabolic health indicators, and biomarkers of dysbiosis and inflammation differ among various studies. Thus, the results across different studies are not always comparable.

To address these limitations, large-scale, long-term clinical trials involving diverse human populations are essential. These trials should aim to evaluate the efficacy and safety of various microbiota-targeting interventions, specifically in older adults. Moreover, standardized protocols and methodologies are necessary to ensure that the results are comparable across different studies and would involve uniform criteria for participant selection, intervention dosages, and outcome measures. Additionally, personalized medicine, which tailors interventions based on an individual’s unique microbiota profile, genetic background, and lifestyle, is essential for optimizing outcomes and transcending generalizations. However, this approach requires advances in metagenomics, metabolomics, and other high-throughput technologies. Finally, while there is a growing body of evidence linking gut microbiota to aging and age-related diseases, the underlying mechanisms remain poorly understood. Future research should aim to elucidate these mechanisms to develop targeted therapies.
